# Optimum utilization of hub energy micro-grids with micro-networking strategy of local biogas productions

**DOI:** 10.1016/j.heliyon.2023.e20995

**Published:** 2023-10-21

**Authors:** Ali Jabbary, Reza Noroozian, Gevork B. Gharehpetian

**Affiliations:** a Department of Electrical Engineering, Faculty of Engineering, University of Zanjan, Zanjan, Iran; b Department of Electrical Engineering, Amirkabir University of Technology, Tehran, Iran

**Keywords:** Biogas, Flexible operation, Hub, Micro-grids,GAMS

## Abstract

In this paper, the optimal utilization of local biogas production in multi-energy systems is investigated. The micro-grid policy of local biogas production, to supply the variable demand of natural gas and also to convert it into electrical energy required by an energy hub micro-grid, along with other sources of scattered energy production and national electricity and gas networks, is one of the main goals of this paper. The multi-factor intelligent optimization method is considered to investigate the non-linear and heterogeneous optimization problem in the IEEE standard 33-bus energy hub microgrid. Most of the main indicators of interest are economic savings and minimization of operating costs. The multi-objective optimization method has been chosen as a method to solve the aforementioned non-linear and heterogeneous problem. Simulation has been done in GAMS software and the results of optimal load distribution of electric energy and natural gas supply for selected energy microhub have been explained and compared. The final results show a significant reduction of 46 % (2.8 thousand dollars per day) in operating costs. Significant reduction of energy flow between local network microhubs by 39.4 % is also one of the optimization results studied. Also, the Island performance of the local energy microhub has been simulated with the desired strategy. The cost of local energy microhub energy supply in island mode has decreased by 34 % (1.4 thousand dollars per day) compared to normal mode.

© 2022 Published by.

## 1 Introduction

**Fig. 1:**
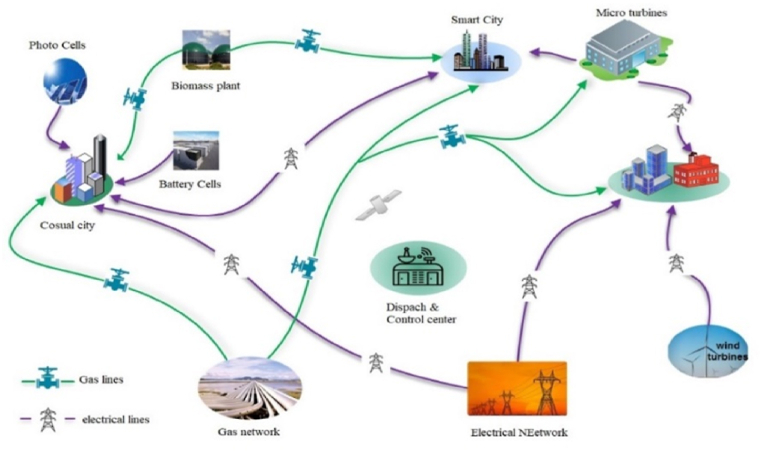
An example of a multi-energy system with electricity and gas feed.

The energy dilemma has been one of the biggest problems for humanity in the past century. Energy security is now a major concern for all nations in the world due to the increase in global demand for various energy sources, the scarcity of fossil fuels, and environmental pollution. Its significance has grown as a result of the intimate connection between energy and economic, social, environmental, and security factors. Heating, cooling, and even natural gas networks share many similarities with electric networks in terms of network losses, low efficiency, pollution, etc. [1]. On the other hand, the management and planning of energy systems have always been done separately. And even when numerous energy networks are present or not, there are still significant losses from frequent or unnecessary transformations of energy carriers. Therefore, all energy must be provided through energy systems, not just electrical energy [2]. In this regard, focusing on the function of multi-energy systems, in which various energy carriers interact and communicate with one another in an optimal manner, is a crucial strategy for achieving sustainable energy systems. Increased economic and environmental benefits, including higher system dependability, lower operating costs, less fuel usage, and lower greenhouse gas emissions, can result from MES performing at its best [3]. However, in order for these systems to operate well, there is a requirement for an ideal integrated management framework that can manage the system's many components in an ideal manner. Uncertainty of load, energy price, production rate of distributed production sources are among the challenges of every microgrid connected to a multi-energy network. The presence of energy storage can be considered as a very effective tool in reducing energy costs and reducing network risk. In Fig. 1, we see a view of a system fed by gas and electricity network. Each of the micro-grids of this network can be in the form of a simple electricity micro-grid, a smart electricity micro-grid and in a more complete state a smart energy hub micro-grid. The exchange of energy between the aforementioned micro-grid areas is not logical considering their long distances and the issue of losses of energy carriers such as heat, cold or hydrogen. But the intelligent management of each energy hub micro-network for several energy carriers in the hub itself is very important in accordance with the overall energy price and also the optimal dispatch of the active gas and electricity distribution network [4].

**Fig. 2:**
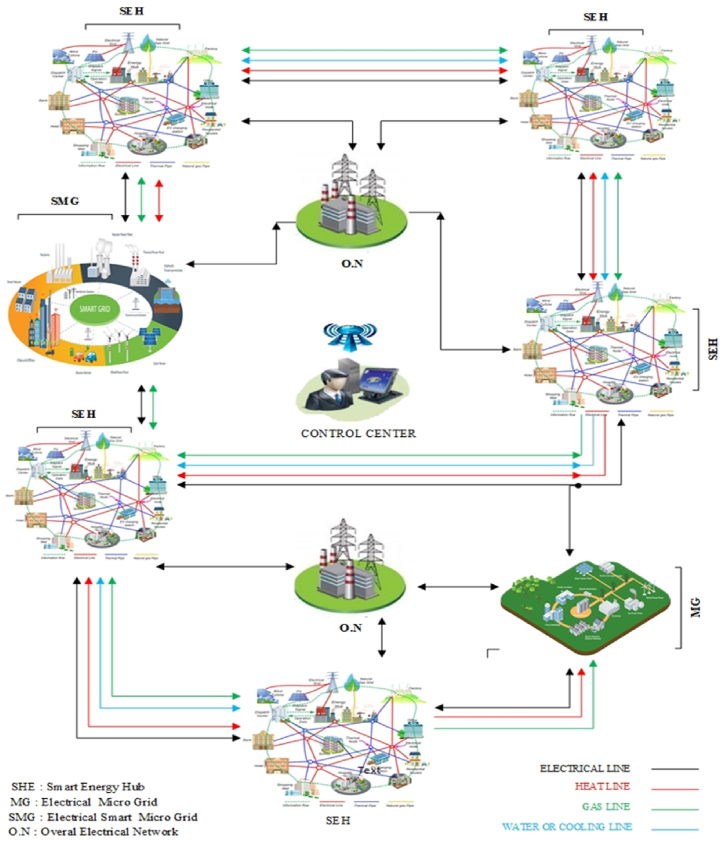
A sample of connection and energy management in a centralized energy network.

**Fig. 3:**
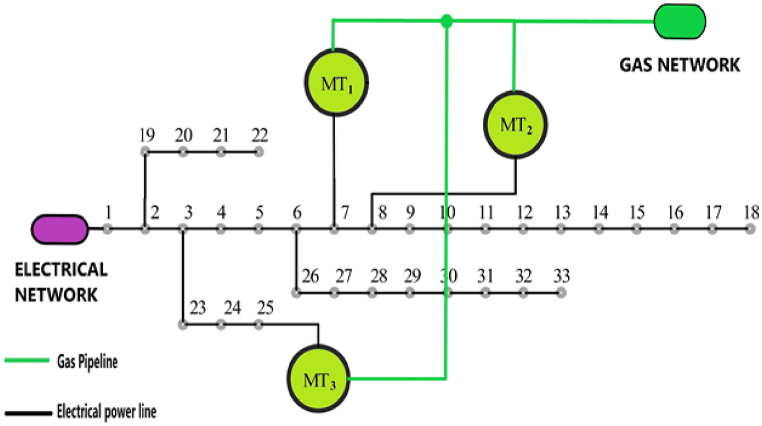
33-bus standard circuit in connection with scattered production sources and the national gas supply network.

Basically, an energy microhub can be operated as a simple or smart microhub, depending on the level of development and investment level. In a smart microhub, important issues such as load flexibility, energy conversion management and its storage, multi-level intelligent multi-objective optimization should be studied. Also, in a vast energy network, connecting the various types of micro-hubs mentioned and managing the local and general energy markets is one of the big challenges of the energy hub [[4], [5], [6]]. Fig. 2 provides a good descriptive example of connecting various technologies of a multi-energy network with multi-agent management of the central operator for optimal use of the entire network. As you can see, in this figure, some local micro-grids are defined only as an electric energy due to the lack of development, and in the next step, they are defined as smart electric micro-grids. This is while there are smart energy hub micro-grids with energy communication channels in this network. Some micro-grids only use electricity, some use electricity and gas, and some exchange 4 components of energy. Of course, the exchange of heat or cooling for close microgrids is economically justifiable in terms of geographical distance [5] (see Fig. 3).

Two types of control strategies can be investigated in the optimal management of such a network. Control within the energy hub and integrated control of the network. From the point of view of the electricity market, each microhub is interested in meeting its energy needs at the lowest cost, participating in the process of buying and selling energy (depending on the amount of domestic production resources) and making maximum profit. In this paper, the optimal economic exploitation of the local energy hub microgrid in connection with the national electricity and gas energy networks has been studied.

## 2 Biogas and biomass power plants

Biogas is a gaseous mixture rich in methane, which is obtained from the anaerobic fermentation of some sources, the most important of which are animal waste and urban sewage sludge, in general, sources of biogas production are organic waste (livestock waste, urban and industrial sewage …), agricultural products (sweet corn, grapes, sunflower, wheat, sugar beet, etc.) and other organic raw materials (glycerin, etc.) [5]. Biogas power plants are of great importance in this study because they can produce electricity and methane gas at the same time. Due to its high calorific value, biogas can be used as a clean and available fuel for cooking and hot water production. Also, with a little change, diesel engines will be able to produce electricity using biogas. The generated electricity can be used to provide lighting. A very important point in these power plants is the possibility of storing the produced gas in the relevant tanks in this power plant. Currently, more than 21 million units of these systems have been installed in the world [6]. In reference 6, appropriate information is provided in terms of estimating the biogas produced and storable for the standard network. In this reference, the goal of reducing energy supply costs in the form of natural gas and electricity for the 33 bus standard network is considered. Despite the consideration of biogas resources in the form of local injection, a gas storage has been considered, and the presence of other sources of scattered electricity generation has been considered less. In reference 8, a good review has been done for the presence of biogas sources alongside scattered production sources, but it is more valuable in terms of controlling the local network and there has not been a review for costs and optimal energy load distribution. In reference 9, the day-to-day performance of biogas sources for the daily storage system is simulated and the amount of electricity produced to the power grid is studied. In this reference, the presence of other gas sources, storage systems from the national gas network are not included. Reference number 10 has presented a suitable multi-objective intelligent simulation for optimal load distribution of a multi-energy system with emphasis on the effect of energy storage. In this reference, it is also considered in a small microgrid in terms of the heat energy utilization system. In reference number 11, a valuable optimization has been made in terms of reducing the costs of connecting biomass sources in a wide area. In this reference, the management and reduction of costs in the presence of the power grid and the possibility of converting these two types of energy have not been investigated. Reference 12, a valuable analysis of the presence of both electricity and gas energy networks with the simultaneous presence of scattered production sources such as wind turbines and solar cells and even the electricity storage system in the form of batteries has been done. This source is considered the national gas network with simple piping and normal connection point to gas power plants, and biogas sources are not included. This reference has emphasized more on the performance of the proposed optimization algorithm. In reference 18, the optimal performance of a multi-energy microgrid has been modeled and simulated. In this reference, despite the study of the heat energy carrier, the effects of energy storage in all three types of studies have been less discussed. In reference 29, a hub micro-network is connected to the network of 34 standard buses. Gas piping is not included in this reference.

## 3 Local biogas resource utilization modes

Figure [3] shows the schematic of the standard 33-bus network next to scattered production sources such as solar cells, wind turbines and batteries. Also, the pipeline route of natural gas supplied through the national gas network has been determined to supply the gas required for 3 gas power plants [7].

Depending on the level of development of the studied area and the type of piping used in the natural gas network, two types of access for biogas production can be considered. We will examine them further.

### 3.1 injection mode from any bus (point)

**Fig. 4:**
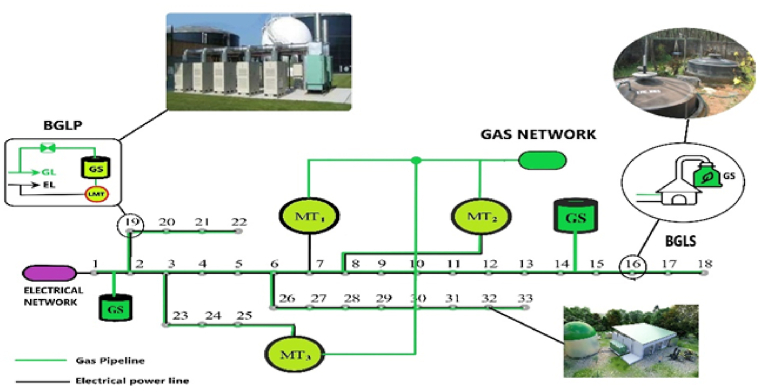
Schematic of the injection system from each local biogas bus and how to connect it to the gas supply network.

In most of the networks, natural gas pipelines have reached the feet of individual consumers. In this design, each Bus is connected to the adjacent Buss by local gas piping. In addition to the consumption of natural gas, each Bus will have the ability to inject the produced gas into the pipeline network with local compressors if it has biogas production. Fig. 4 shows the schematic of the local network for the point-to-point injection mode. In Bus No. 16, only the production and injection of natural gas is possible, while in Bus No. 19, in addition to the injection of natural gas, due to the presence of local microturbines, it will also be possible to inject electricity.

The gas stored in such a system for microgrid islanding conditions can greatly increase the reliability of electricity supply and its reliability. Despite having a very high investment cost, this plan will have significant technical benefits.

#### 3.1.1 regional injection of biogas

In the previous plan, the most important primary limitation will be the high cost of biogas storage equipment in each Bus as well as piping from Bus to Bus. In the less expensive plan, by removing the general piping, it is possible to imagine the piping and even the local power plant only for areas with high production volume. The biogas produced in this plan is accumulated in an area and used for the consumption of large gas power plants, local power plants or direct connection to the national gas network.

**Fig. 5:**
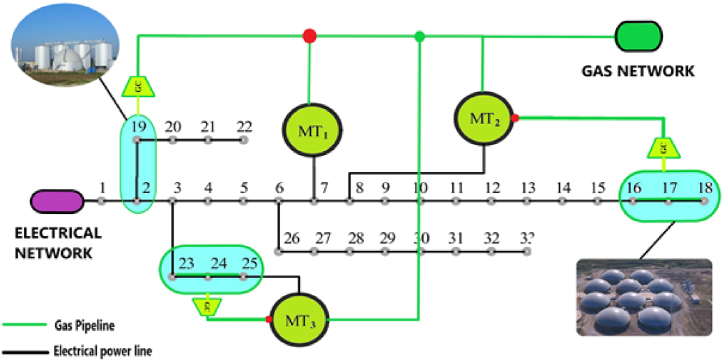
Regional injection of biogas into the gas network or nearby power plants.

Fig. 5 provides a good descriptive example for this exploitation model. As you can see, it is possible to produce, store and inject local and industrial biogas in only 3 areas. Natural gas piping and the possibility of consuming local biogas from other tires are not possible.

## 4 Modeling and problem formulation

Basically, two categories of formulations are necessary to simulate the optimal conditions of energy microhub. The first category is related to the constraints of equality and inequality governing the electricity network and the gas network, which are known as the constraints of the economic optimal load distribution equations. These limitations and their detailed explanation are presented and discussed in Ref. [6]. The second category is related to the cost functions of electricity and gas network equipment. Different authorities have provided different formulations in this case. Reference [8] has had a good review on the work done in this field. Considering that the aim of this paper is the optimal exploitation of local biogas resources along with other sources of scattered production in terms of economy and cost, therefore, the formulation of the network elements studied in Fig. 7 as the local network studied in this paper, has been examined in terms of cost. Equation (1) expresses the total cost function of the studied local energy microhub.

(1) $$C_{t}^{MEG}=C_{t}^{PV}+C_{t}^{W}+C_{t}^{FC}+C_{t}^{MT}+C_{t}^{GASN}+C_{t}^{bat}$$

### 4.1 Electrical grid formulation

In this study, the cost functions related to distributed electrical generation sources, in the form of batteries, wind turbines, microturbines, Photovoltaic, and fuel cells, have been considered. The cost functions of scattered electrical generation sources in this network are presented in Table 1. A complete description of the cost functions and related constraints is provided in reference [7]. The formulation (1), (2), (3), (4), (5), (6), (7), (8), (9), (10), (11), (12), (13) used in this table has stated the costs related to investment costs, operation and maintenance costs based on the production capacity of each unit.

**Table 1:** formulation of electrical generation sources cost functions [7].

Auxiliary relations	COST FUNCTION	D.G	NO
(3) $P_{t}^{W}=C_{p}\left(V_{t}\right)N,\forall t\in T$	(2) $C_{t}^{W}=\alpha^{W}\times p_{t}^{W}+\beta^{W}\times p_{t}^{W}+\gamma^{W}\times p_{t}^{W}\forall t\in T$	Wind Turbine	2,3
(5) $C_{fuel}^{MT}=C_{gas}\sum_{t}\frac{P_{t}^{MT}}{\eta_{t}},\forall t\in T$ (6) $C_{m}^{MT}=\sum_{t}K_{m}P_{t}^{MT},\forall t\in T$ (7) $$\begin{array}{c} C_{em}^{MT}=\sum_{t}\sigma 10^{-3}\alpha^{MT}+\beta^{MT}p_{t}^{MT}+\\ \gamma^{MT}\times \left(P_{t}^{MT}\right)+\lambda \;\exp\left(\xi P_{t}^{MT}\right) \end{array}$$	(4) $C_{total,t}^{MT}=C_{fuel,t}^{MT}+C_{m,t}^{MT}+C_{em,t}^{MT}$	Micro Turbine	4,5,6,7
(9) $P_{t}^{BT}=C\left(B\right)*N,\forall t\in T$	(8) $C_{t}^{BT}=\alpha^{BT}\times p_{t}^{BT}+\beta^{BT}\times p_{t}^{BT}+\gamma^{BT}\times p_{t}^{BT}\forall t\in T$	Batteries	8,9
(11) $P_{t}^{PV}=G_{t}^{\beta }\eta AN,\forall t\in T$	(10) $C_{t}^{PV}=\alpha^{pv}\times p_{t}^{PV}+\beta^{pv}\times p_{t}^{PV}+\gamma^{pv}\times p_{t}^{PV}\forall t\in T$	Photovoltaic	10,11
(13) $P_{t}^{FC}=V_{t}I_{t}N,\forall t\in T$	(12) $C_{t}^{FC}=\alpha^{FC}\times p_{t}^{FC}+\beta^{FC}\times p_{t}^{FC}+\gamma^{FC}\times p_{t}^{FC}\forall t\in T$	Fuel Cell	12,13

### 4.2 Natural gas network formulation

A gas source, also known as a gas well, a pipeline, compressors, interconnection, storage, and a gas load are all components of the gas network. The equations related to natural gas flow and biogas sources are presented in Table 2. A complete description of the cost functions and related constraints is provided in reference [9]. In this table, all the costs related to investment, piping, natural gas emissions are presented with proper formulation (14–29).

**Table 2:** Formulation of natural gas network [9].

Formulation	Parameter NO	Formulation	Parameter	NO
22 $3650\sum_{i}f_{pi}f_{Ti,steam}f_{Ti,gas}K_{i}^{0.8}C_{HE\left(HRSG\right)}$		14 $C_{HE\left(HRSG\right)}+C_{\text{piping}}+C_{gas}C^{\text{GASN}}$		
23 $130{\left(A_{HE/0.093}\right)}^{0.78}C_{HE}$		15 $0.0971\left(P_{i}/30\right)+0.9029f_{PI}$		
24 $442{\left({\overset{\cdot }{W}}_{\text{pump}}\right)}^{0.71}1.41f_{h}C_{pump}$		16 $1+\exp\left(T_{\text{out},steam}-830/500\right)f_{Ti,\text{steam}}$		
25 $$\exp\left\{\begin{array}{l} 10.158+0.1003\left[\mathrm{Ln}\left(A\right)\right]\\ +0.04303{\left[\mathrm{Ln}\left(A\right)\right]}^{2} \end{array}\right\}C_{\text{dryer}}$$		17 $1+\exp\left(T_{out,steam}-990/500\right)f_{Ti,\text{gas}}$		
26 $760V^{0.22}C_{shs}$		18 $Q_{i}/DT_{\text{LM},i}K_{i}$		
27 $0.04HRSG_{Cost}C_{\text{HRSGstack}}$		19 $11820\sum_{j}f_{pj,\text{steam}}K_{\text{piping}}$		
28 C=GT[(−98.328LN(WGT)+1318.5)WGT]CGT		20 $0.0971\left(P_{j}/30\right)+0.9029f_{pj}$		
29 $C_{COMP}=91562{\left[\frac{W_{COMP}}{445}\right]}^{0.67}C_{\text{COMP}}$		21 $685m_{\text{gas}}^{1.2}C_{gas}$		

## 5 Problem solving strategy

**Fig. 6:**
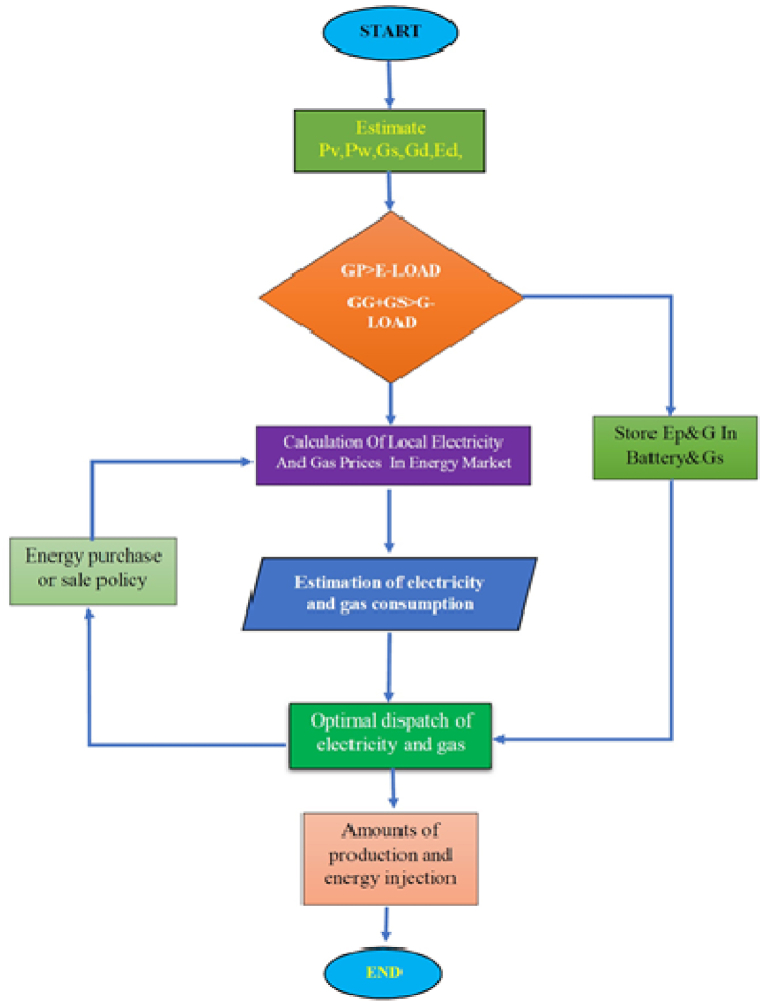
dispatch strategy overview.

Just as the power grid tries to feed the electrical microgrids in an island manner and with the lowest cost, the Hub Energy microgrid also tries to inject gas from the grid with the least amount, relying on renewable resources as well as gas production and storage resources. In addition to the electrical loads, the gas supply also supplies the gas loads. This issue is related to the number of gas and electricity exchange points in the energy hub micro-grid (such as gas power plants), electricity storage sources, gas storage sources, local energy prices, technical indicators of electricity and gas transmission such as losses, electricity flow rate and Gas in the network as well as distribution of the economic load of energy in the energy hub will be a non-linear and complex issue. To solve this problem in an energy hub microgrid, the priority is to optimally use the scattered production resources of each energy hub and optimal energy storage in the studied microhub to reduce energy injection [[10], [11], [12]]. The next priority is to reduce all the cost functions and distribute the economic load inside the desired microhub. To optimally solve this problem, we will need non-linear multi-objective optimization Fig. 6 shows the optimization strategy of energy economic load distribution.

Gas produced and stored in gas tanks from biomass, due to its long production and storage time, will not completely eliminate the need for the local network from national gas consumption. On the other hand, it is not possible to store a permanent surplus of this gas beyond the capacity of the storage tanks. Here, the policy of using this gas for its optimal benefit will be very important. For example, if the gas produced and stored is considered only for emergency situations such as island conditions of the local network, the market operator cannot use this energy source in the energy market more than a certain limit. In this policy, indicators such as network stability and Reliability will be a top priority. But in another policy, the supply of permanent energy to the network with the lowest cost or the peak of the national network has a more important priority, and the market operator in the National Dispatch Center will be able to use all the energy reserves in the energy reservoirs. In this paper, considering the role of biogas resources in the optimal operation of the network, it is possible to use all the gas reserves in the storage tanks, and the market operator will be able to use part or all of it in the energy price and need. Use it to improve operating conditions and reduce costs. Although due to the permanent production of biogas resources, policies with the perspective of long-term planning can be studied. As can be seen in the strategy drawn in Fig. 5, in the first step, the electricity production situation is checked in terms of feasibility and available power of scattered production sources according to the weather conditions and related restrictions. On the other hand, the amount of gas available in the gas storage sources is obtained by considering the national gas price and the gas consumption demand of the local network. Considering that different seasons of the year have a direct effect on gas consumption policy, this study can be studied for three seasons: summer, spring and winter. For example, gas consumption peak is in winter and electricity peak is in summer. Therefore, the price of electricity and gas will vary greatly depending on the different seasons of the year. In this paper, according to the main goal of the paper to investigate the role of biogas, the winter season has been chosen for the study. In this season, the price of natural gas is higher than in any other season of the year, and even many gas power plants face serious restrictions to produce electricity. In the next step, if the electric power produced by the distributed production sources in the studied micro-hub is more than the amount of power required by the demand load, this power is directly stored and also due to the high price of natural gas, the production of gas power plants, is limited. In local networks with high production and intelligent consumption management, it will be possible to sell energy in the energy market. In the next step, the multi-objective nonlinear optimization problem for the optimal allocation of energy production and distribution in the local network is investigated by GAMS software. The results obtained at this stage are intended to reduce existing costs and spread the economic burden. At this stage, the optimal production power values of local power plants and scattered production sources are obtained along with the economic use of electricity and gas energy storage resources. The available biogas reserves are also used for electricity generation or direct consumer consumption according to the conditions of energy consumption optimization.

**Fig. 7:**
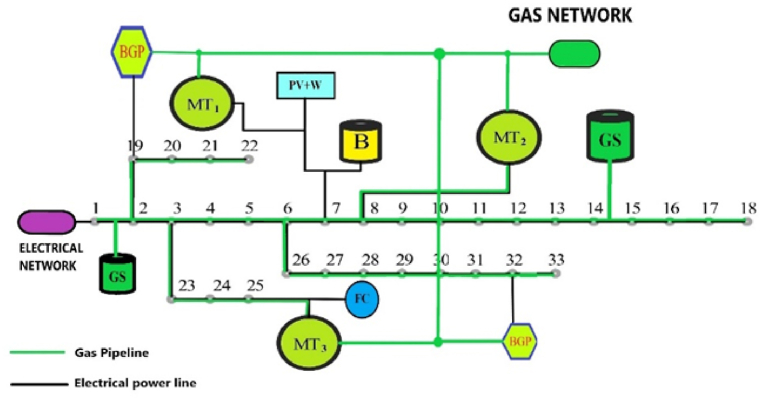
IEEE standard 33 bus network along with gas network and distributed generation sources.

## 6 Case study

To study the strategy discussed in paragraph 6, in this part the network of figure (7) is considered. The two large biogas power plants in the 19th and 32nd streets have the possibility of transferring electricity to the local grid, and they also have the possibility of producing gas and injecting it into the gas grid. The specifications of the relevant power plants are provided in Ref. [13]. Also, microturbines 1 to 3 are placed in buses 7, 8 and 23, respectively. The possibility of injecting natural gas into each of the buses of the local network is considered. Although all or at least some of these Buss are capable of producing local electricity with the help of small microturbines, depending on their development, here only the gas production of these sources is considered. Depending on the conditions of the energy market, based on the priority of optimization, the produced gas can be converted into electricity in gas power plants or used to meet the demand for consumed gas. The policy of using natural gas produced for electricity generation or consumer gas demand is decided according to the market conditions and the optimization algorithm for each moment.

**Fig. 8:**
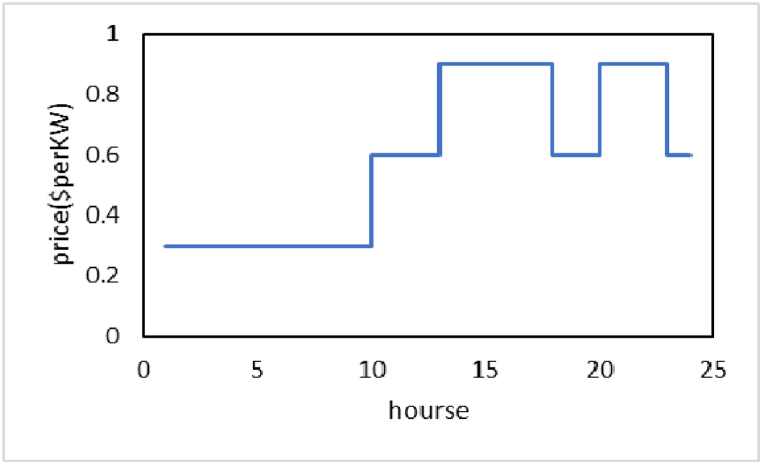
Price of electricity in 24 h.

**Fig. 9:**
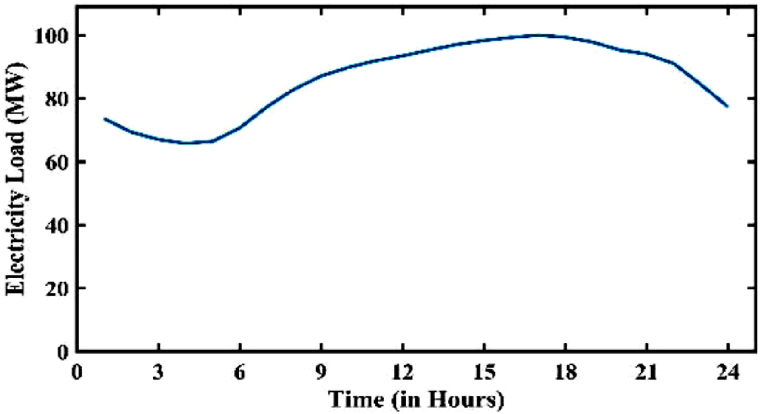
Load changes in 24 h.

Considering the peak loads in the winter season for both electricity and gas factors compared to other seasons, in this paper the winter conditions have been examined. Siemens SGT-A65 gas turbines are the type of microturbines utilized here. They have a 65.3 MW combined cycle power production capacity at 50 Hz. 53.6 % is the highest possible operating efficiency. The used battery bank consists of 8 lithium-ion batteries with a nominal capacity of 10 MWh, which has a total storage capacity of 80 MWh. Electric demand and gas consumption, market price are presented as follows [[12], [14], [15]]. In the simulation, electricity is traded according to a Time of Use price (TOU) policy on the main system, also known as the national grid. A lower price, a regular price, and a higher price are the three intervals for the trading price. As shown in Fig. 8. The first day's events take place between midnight and 6 a.m. The second break is between 9:00 and 11:00 and 17:00 and 19:00. The third break is between 12:00 and 16:00 and 20:00 and 24:00. Additionally, there is a price difference between buying and selling the main grid (see Fig. 9).

In figure (9), the biogas stored for one month on each bus of the standard network is estimated. As can be seen, Buss 24 and 25 have more capacity to produce and store biogas. This issue depends on indicators such as population and available technology [28].

**Fig. 10:**
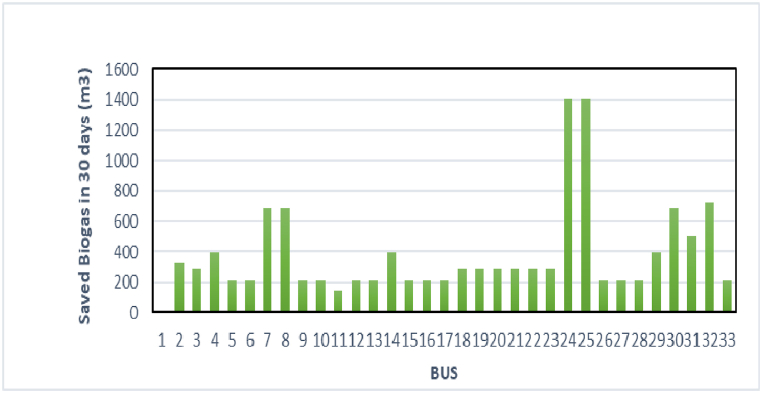
Biogas stored in the busses of the studied network for a period of 30 days.

The problem of estimating biogas production for each Bus in the local network is a complex and uncertain issue. And it is possible only in the case of installation and operation of the devices for producing, storing and converting biogas in each Bus. Since in this paper, the effects of these sources in networking have been studied, therefore, this issue has been investigated with a logical estimation method. The amount of biogas produced in each Bus is directly dependent on the population living in the geographical environment of that Bus. Because the production of human excreta and sewage converted to produce biomass is directly dependent on the people of that area. The more the population of a region is, the more biogas production of the region can be definitely said. On the other hand, the amount of electricity consumption in each Bus, assuming that the use of network Buses is mainly domestic, will be related to the population of that Bus. In other words, the level of biogas production of that Bus can be understood from the consumption demand of each Bus. In principle, a coefficient can be used to estimate the relative consumption demand (MW) of each Bus to its produced biogas(M^3^). This coefficient will directly depend on factors such as the type of load (domestic, industrial, etc.) and the level of development. Given that the network The area under study of the paper is (The 33-Bus standard micro-network from the distribution network of New York, USA) and the load values of each Bus are known, the relative and default estimate for this network is presented in the form of a diagram in Fig. 10 of the paper. Another method to estimate useable biogas from the population density of Buss is to multiply the standard electricity that can be produced per person during the day and night.

**Fig. 11:**
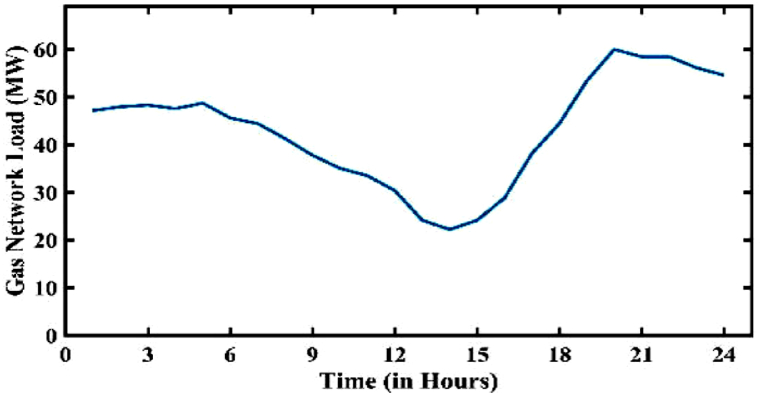
Load curve of the gas network (excluding MT).

**Fig. 12:**
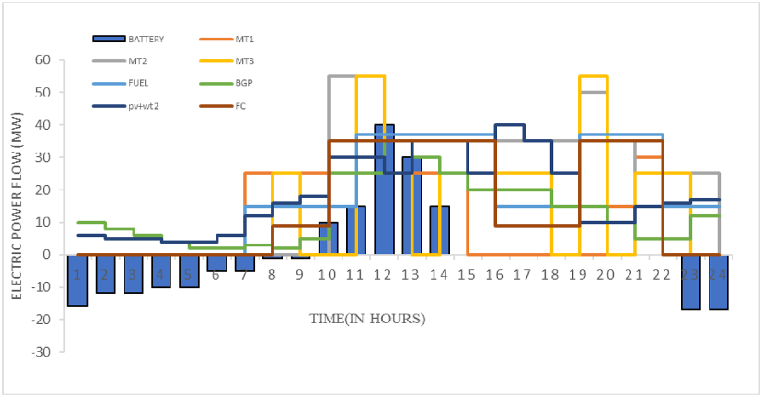
Dispatch simulation results for the electrical network.

The load and price of electricity generally follow the same pattern in the electricity network. In a similar vein, Fig. 11 depicts the pattern of gas network load from a typical day, which shows the household load of the gas network (see Fig. 12).

The electrical parameters of the studied network are given in Table 3.

**Table 3:** Electrical parameters of study case.

SOURCE DESCRIPTION	SOURCE DESCRIPTION
Micro Turbine	3 × 66 MW/H
Fuel cell	40 MW/H
Wind Turbine	50 MW/H
PV	100 MW/H
Grid (±max)	250 MW/H
Load (max)	250 MW/H
Battery bank	80 MWH

The battery bank consists of eight lithium ion-based battery units, each with 10 MWh capacity, or 80 MWh in total. The natural gas network components’ parameters are given in Table 4.

**Table 4:** Gas network parameters.

SOURCE	DESCRIPTION
Gas source 1	**13,800 M3/H**
Gas source 2	**10,200 M3/H**
Gas load (max)	**6000 M3/H**
Gas MT demand (max)	**31,200 M3/H**

## 7 Results and discussion

The studied network is simulated for two normal states and an island state. The numerical results obtained in both cases have been compared.

### 7.1 Normal network) connected to national electricity and gas networks(

The results of optimal load distribution for 24 h based on the multivariable optimization strategy expressed in figure (12) ar e shown. Computer system used for simulation is Core i7, CPU: 4 GB, RAM: 64 Bit. The contribution of each of the scattered production sources in the local grid electricity supply and also the amount of production of power plants are shown along with the amount of energy purchased from the national electricity grid. For low-load hours, electricity is stored in batteries from 00:00 to 08:00 in the morning. As the load demand increases, the use of stored energy is intelligently increased. Also, the electricity produced from the excess natural gas of the local network is also considered as electricity from biogas sources. The production of biogas resources in the form of electrical energy has been done after supplying the gas demand of the local network.

**Fig. 13:**
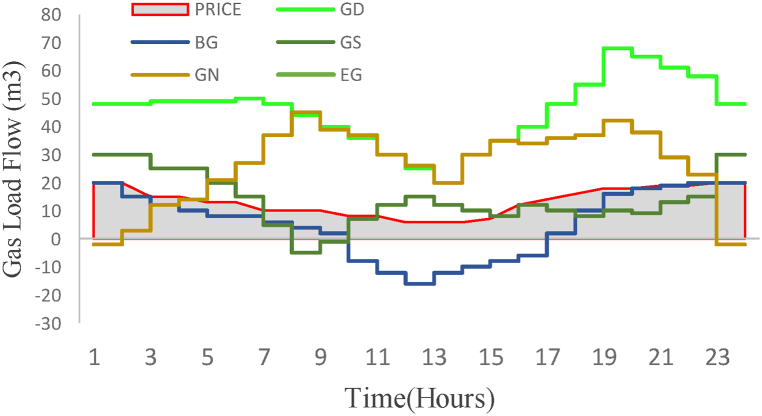
Dispatch simulation results for the gas network.

**Fig. 14:**
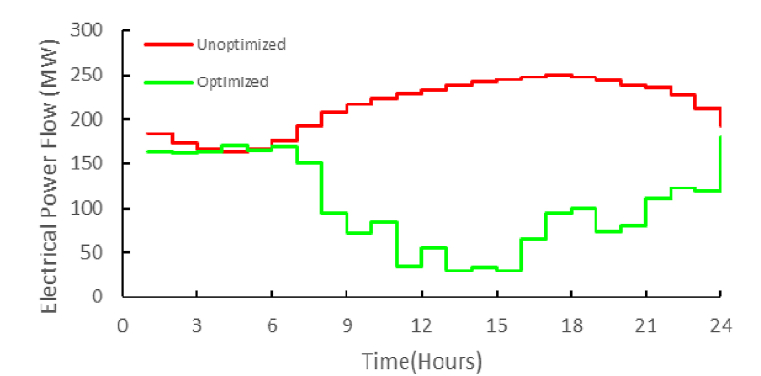
Sale and purchase for the main grid.

Also, the results of optimal natural gas load distribution simultaneously with electric load distribution 24 h a day are shown in Fig. 13. In this graph, the demand for consumed gas (GD), natural gas produced from biogas sources (BG), gas stored in natural gas storage tanks (GS), gas purchased from the national gas network (GN) and gas converted into energy Electricity (EG) is shown in the optimization process next to the current price of gas in the energy market. Also, the amount of financial savings during optimization conditions in the complete stages of production to energy consumption is estimated at 508 thousand dollars in one night (see Fig. 14).

To compare the optimized conditions with the pre-optimization conditions and to examine the effect of scattered production sources, especially the effect of biogas sources on the degree of independence of the local network and its peak power, figure (14) is presented for electric energy is shown. Table 5 shows the values of the calculated costs for four different operating modes of the studied microgrid. The first case is related to the independent supply of electricity and gas by two independent operators (similar to very traditional networks). In this case, the energy conversion and storage potentials in the microgrid have not been taken into account, and basically the energy purchase prices have been calculated. The second case is related to the non-optimized mode. The non-optimal state means that the network operator, regardless of the microgrid's economic dispatch, energy conversion and storage potentials, the economic priority of using local electricity and gas sources, and the current price of energy, and only with the strategy of permanent energy supply, buys energy. Electricity and gas from the national grid. The third item is the total cost of supplying electricity and gas energy demand of the local microgrid with optimization, considering the investment costs of local biogas production, storage, distribution and conversion equipment. The fourth item shows the optimized total cost, without the investment costs of the biogas network and related equipment.

**Table 5:** Daily energy savings.

	Case 1	Case 2	Case 3	Case 4
Total cost($)	398212	381360	353180	204568
Cost savings($)	0	16852	28180	148612

Table 6 shows the gas power plants in the process of feeding the network load. The amount of cost savings is estimated for each mode. It can be seen that in the third case, the daily cost has decreased by 28.18 thousand dollars. This problem after the return of capital of biogas equipment reaches the daily value of 148.6 thousand dollars, which is equivalent to 46.3 % saving in energy supply costs compared to the state without optimization.

**Table 6:** Working hours of gas turbines in 24 h.

UNIT	HOURS [[1], [2], [3], [4], [5], [6], [7], [8], [9], [10], [11], [12], [14], [15], [16], [17], [18], [19], [20], [21], [22], [23], [24], [25]]
**1**	0 0 0 0 0 0 0 1 1 1 1 1 1 1 1 1 0 0 0 1 1 1 1 0
**2**	0 0 0 0 0 0 0 0 0 0 1 1 1 1 1 1 1 1 0 1 1 1 1 1
**3**	0 0 0 0 0 0 0 0 1 0 1 1 0 1 1 1 1 1 0 1 0 1 1 0

**Fig. 15:**
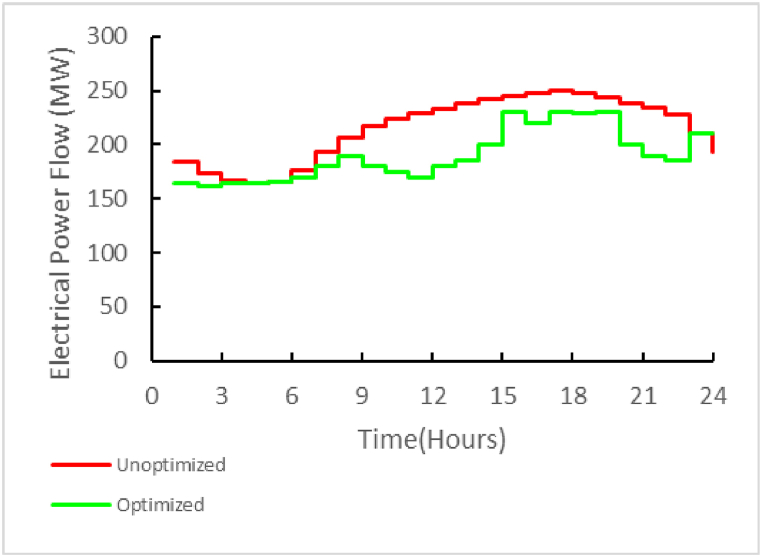
Effect on the regional grid.

Although the optimization strategy used focuses on reducing local network costs, the results of this optimization have a good effect on reducing the transmission load between two areas as well as peak performance. This issue can be seen in Fig. 15. Simple mathematical calculations indicate a reduction of 348 MWH in the flow of electrical energy between the grids in 24 h. This value is equivalent to 39.4 % compared to the non-optimized state.

### 7.2 Island operation mode

**Fig. 16:**
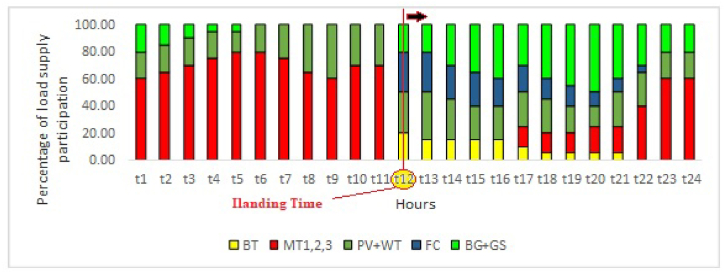
The results of optimal load distribution between distributed generation sources of the studied local network in island mode.

In this part, the investigation of the effect of biogas sources on the behavior of the studied network in terms of the reliability index of the network is considered. Fig. 16 shows the results of optimal load distribution between distributed generation sources of the studied local network in island mode in terms of generation percentage. The most important goal is to supply 100 % of the consumed electric load of the network with the lowest cost in addition to supplying the demand for consumed gas. In this scenario, it is assumed that the disconnection of the local network happened at the peak of the day, that is, at 12:00 noon. And this situation continued for the next 24 h. In this scenario, the connection of the gas network to the national grid is assumed to be stable, and as a result, there is a lack of electrical energy supply through the consumption of national grid gas and biogas sources. As it can be seen, the biogas resources of the network in the island mode play an important role in providing electrical energy until the time of their reserves. With the completion of charging batteries and biogas reserves, the supply of the network load is the responsibility of the national gas network through the existing microturbines. Considering the winter season and the high price of natural gas, this issue will lead to a high cost in the final hours of the island mode. Also, any restrictions on gas supply in this scenario, especially in the late hours, will lead to energy shortages and blackouts imposed on network consumers.

For the island mode, in non-optimal conditions, the stability of the grid without blackout of electricity and gas consumption, for 24 h, requires a cost equal to 415,325$. This cost is due to winter conditions and high gas prices. While this cost for the optimal mode is $274,114. which reports cost savings equivalent to 141,211 dollars per day. This reduction is equivalent to 34 % of the total cost.

## 8 Conclusions

In this paper, while examining the utilization modes of local biogas production, the estimation of biogas production in each tire, the formulation of investment costs and the exploitation of local biogas resources have been examined. The aforementioned productions have been examined along with other sources of scattered electricity and gas energy production in the standard 33-bus IEEE network connected to national energy networks. To simulate optimal operating conditions from the point of view of the cost index, multi-objective optimization has been used by GAMS software and its results have been examined and compared. In short, the results of the paper can be presented as follows.

1. The energy distribution strategy of an energy hub microgrid in its connection to the natural gas and electricity grid, along with scattered production sources and local natural biogas production, is formulated in the form of a multi-objective nonlinear optimization problem in gams software. It is simulated.
2. In the aforementioned formulation, the costs of pollution treatment or fines for the emission of toxic gases, the costs related to the storage and maintenance of local biogas and the cost of compressing local gas to the natural gas network are also included.
3. Savings in the cost of optimal operation in the normal state is 28.18 thousand dollars on a daily basis and after the investment return and deduction of the investment cost, 146.8 thousand dollars has been achieved on a daily basis. These values are respectively 7.3 and 46.3% improvement compared to the traditional operation mode of Hub Energy network.
4. The strategy used has not only led to optimal dispatch and daily cost reduction, but has also led to a significant improvement in the technical indicators of the network, including the reliability and reliability of the micro-network.
5. The flow of electrical energy between the network in the optimized mode has decreased to the amount of 348 MW h (39.4 %) for every 24 h compared to the non-optimized mode.
6. In the island mode (local microhub power cut scenario), the cost savings of providing gas and electricity demand simultaneously for every 24 h is calculated as $141,211 for the optimal mode. which reports a 34 % reduction in the energy supply of the studied microhub under optimized conditions.
7. The investment cost of production and storage equipment and local biogas compressor is high, despite this issue, the improvement of Microhub Energy's operation costs to the amount of 7.3 % is worth noting. This problem is very significant after the return of the invested costs and it reaches the amount of 46.3 % daily reduction in network operating costs.

## Data availability statement

No data was used for the research described in the article.

## CRediT authorship contribution statement

**Ali Jabbary:** Conceptualization, Data curation, Formal analysis, Writing – original draft. **Reza Noroozian:** Conceptualization, Data curation, Methodology, Software, Writing – review & editing. **Gevork B. Gharehpetian:** Conceptualization, Data curation, Formal analysis, Resources, Software, Writing – original draft.

## Declaration of competing interest

The authors declare that they have no known competing financial interests or personal relationships that could have appeared to influence the work reported in this paper.

## Nomenclature

Variable: Description
A: Area of PV panels
$C_{t}^{FC}$: Total social cost of FC at time *t*
$C_{t}^{bat}$: Total social cost of battery at time *t*
$C_{total}^{MT}$: Total social cost of MT at time *t*
$C_{t}^{PV}$: Total social cost of PV at time *t*
$C_{fuel}^{MT}$: Fuel cost for MT
$C_{t}^{W}$: Total social cost of a wind turbine at time *t*
$C_{gas}$: Natural gas price per unit
$C_{P}$: Fuel cost for MT
$C_{m}^{MT}$: MT maintenance cost
$C_{em}^{MT}$: Pollution treatment cost for MT
$I_{t}$: current of Fuel cell stack
$K_{m}$: Maintenance cost constant for MT
N: Units installed Number
$P_{t}^{PV}$: Estimated power generated by PV at time *t*
T: All hours in the year
$V_{t}$: DC voltage between stack of FC at time *t*
$v_{t}$: Velocity of the wind at time *t*
α: Maintenance coefficient
β: Emission characteristic coefficient
γ: Pollution treatment cost coefficient
η: Efficiency of units
ζ: Emission characteristic coefficient for MT
σ: Unit pollution cost
λ: Emission characteristic coefficient for MT
